# Base-resolution methylomes of gliomas bearing histone H3.3 mutations reveal a G34 mutant-specific signature shared with bone tumors

**DOI:** 10.1038/s41598-020-73116-x

**Published:** 2020-09-30

**Authors:** Yuhei Sangatsuda, Fumihito Miura, Hiromitsu Araki, Masahiro Mizoguchi, Nobuhiro Hata, Daisuke Kuga, Ryusuke Hatae, Yojiro Akagi, Takeo Amemiya, Yutaka Fujioka, Yasuhito Arai, Akihiko Yoshida, Tatsuhiro Shibata, Koji Yoshimoto, Koji Iihara, Takashi Ito

**Affiliations:** 1 Department of Biochemistry, Kyushu University Graduate School of Medical Sciences, 3-1-1 Maidashi, Higashi-ku, Fukuoka, 812-8582 Japan; 2 Department of Neurosurgery, Kyushu University Graduate School of Medical Sciences, Fukuoka, Japan; 3 Division of Cancer Genomics, National Cancer Center Research Institute, Tokyo, Japan; 4 Department of Diagnostic Pathology, National Cancer Center Hospital, Tokyo, Japan; 5 Department of Neurosurgery, Graduate School of Medical and Dental Sciences, Kagoshima University, Kagoshima, Japan

**Keywords:** Cancer, Epigenomics, DNA methylation

## Abstract

Two recurrent mutations, K27M and G34R/V, in *H3F3A*, encoding non-canonical histone H3.3, are reported in pediatric and young adult gliomas, whereas G34W mutation is prevalent in bone tumors. In contrast to K27M mutation, it remains elusive how G34 mutations affect the epigenome. Here we performed whole-genome bisulfite sequencing of four G34R-mutated gliomas and the G34V-mutated glioma cell line KNS-42 for comparison with gliomas harboring K27M and no mutations in *H3F3A* and with G34W-mutated bone tumors. G34R-mutated gliomas exhibited lower global methylation levels, similar CpG island (CGI) methylation levels, and compromised hypermethylation of telomere-proximal CGIs, compared to the other two glioma subgroups. Hypermethylated regions specific to G34R-mutated gliomas were enriched for CGIs, including those of *OLIG1*, *OLIG2,* and canonical histone genes in the *HIST1* cluster. They were notably hypermethylated in osteosarcomas with, but not without, G34W mutation. Independent component analysis revealed that G34 mutation-specific components shared a significant similarity between glioma and osteosarcoma, suggesting that G34 mutations exert characteristic methylomic effects regardless of the tumor tissue-of-origin. CRISPR/Cas9-mediated disruption of G34V-allele in KNS-42 cells led to demethylation of a subset of CGIs hypermethylated in G34R-mutated gliomas. These findings will provide a basis for elucidating epigenomic roles of G34 oncohistone in tumorigenesis.

## Introduction

DNA methylation is a major epigenetic modification involved in a wide variety of biological processes and hence various diseases, especially, cancers. Global CpG hypomethylation and focal hypermethylation at CpG islands (CGIs) are well-known hallmarks of cancers^1–3^ and contribute to carcinogenesis via distinct mechanisms: global hypomethylation causes chromosome instability leading to various structural variations, whereas hypermethylation of promoter CGIs suppresses the expression of downstream genes, especially, tumor suppressor genes.

Genetic alterations involved in gliomagenesis have been extensively investigated^4–7^. These alterations include *IDH1/2* mutations and 1p/19q co-deletion, which have served as the bases for the new nomenclature of central nervous system tumors^8^. The association of epigenetic alterations with glioma has also been reported. For instance, *IDH1/2*-mutated gliomas harbor a characteristic methylation pattern defined as the glioma-CGI methylator phenotype and associate with favorable prognosis. Methylation of the promoter of *MGMT*, encoding *O6*-methylguanine-DNA methyltransferase, was demonstrated to be a predictive factor for chemosensitivity to alkylating agents^9^. A recent study demonstrated the utility of methylation profiling in auxiliary diagnosis of central nervous system tumors^10^.

The *H3F3A* gene is one of the two genes encoding histone H3.3, a non-canonical histone variant, and has been established as a major driver gene of malignant gliomas, particularly pediatric and young adult gliomas^11–13^. Histone H3.3 is loaded to transcriptionally active regions in a replication-independent manner and is conserved among various species, ranging from yeast to human^14^. Several cancers bear missense mutations that affect the N-terminal tail of histone H3.3. Two recurrent mutations of *H3F3A*, K27M and G34R/V, have been identified in glioma, whereas G34W/R/L mutations of *H3F3A* are prevalent in giant cell tumor of bone (GCTB) and K36M mutation of *H3F3B* recurs in chondrosarcoma^15–17^.

Previous studies on gliomas revealed that K27M and G34R/V subgroups correlate with distinct clinicopathological features such as disease prevalence, age, tumor location, prognosis, MRI findings, and histopathological diagnosis^11,18^. Distinct patterns of gene expression and DNA methylation, which likely affect disease phenotypes, have also been reported for these gliomas primarily by microarray analyses^12^. Epigenetic mechanisms of tumor development have been well elucidated for the K27M subgroup, which is defined as "diffuse midline glioma, H3 K27M-mutant" in the latest WHO classification^10^. Histone H3.3 bearing K27M substitution traps the Polycomb Repressive Complex 2 (PRC2) to inhibit its histone methyltransferase activity, thereby leading to a global decrease and a local increase of histone H3 trimethylated at its K27 residue^19,20^. Accordingly, EZH2, the catalytic component of PRC2, is a promising target of new molecular targeted therapy^21,22^.

On the other hand, the mechanism behind the contribution of G34 mutations to tumorigenesis remains elusive. As an approach to address the epigenetic mechanisms of action for G34 variants, we conducted whole-genome bisulfite sequencing (WGBS) by post-bisulfite adaptor tagging (PBAT)^23,24^ to delineate single base-resolution methylomes of four G34-mutated glioma cases characterized in our previous study^25^. Comparison of the WGBS data with those of other types of gliomas and bone tumors highlighted a unique methylomic signature shared with bone tumors bearing G34 mutations.

## Results

### Global hypomethylation and exacerbated variation of regional methylation in G34-mutated gliomas

**Figure 1:**
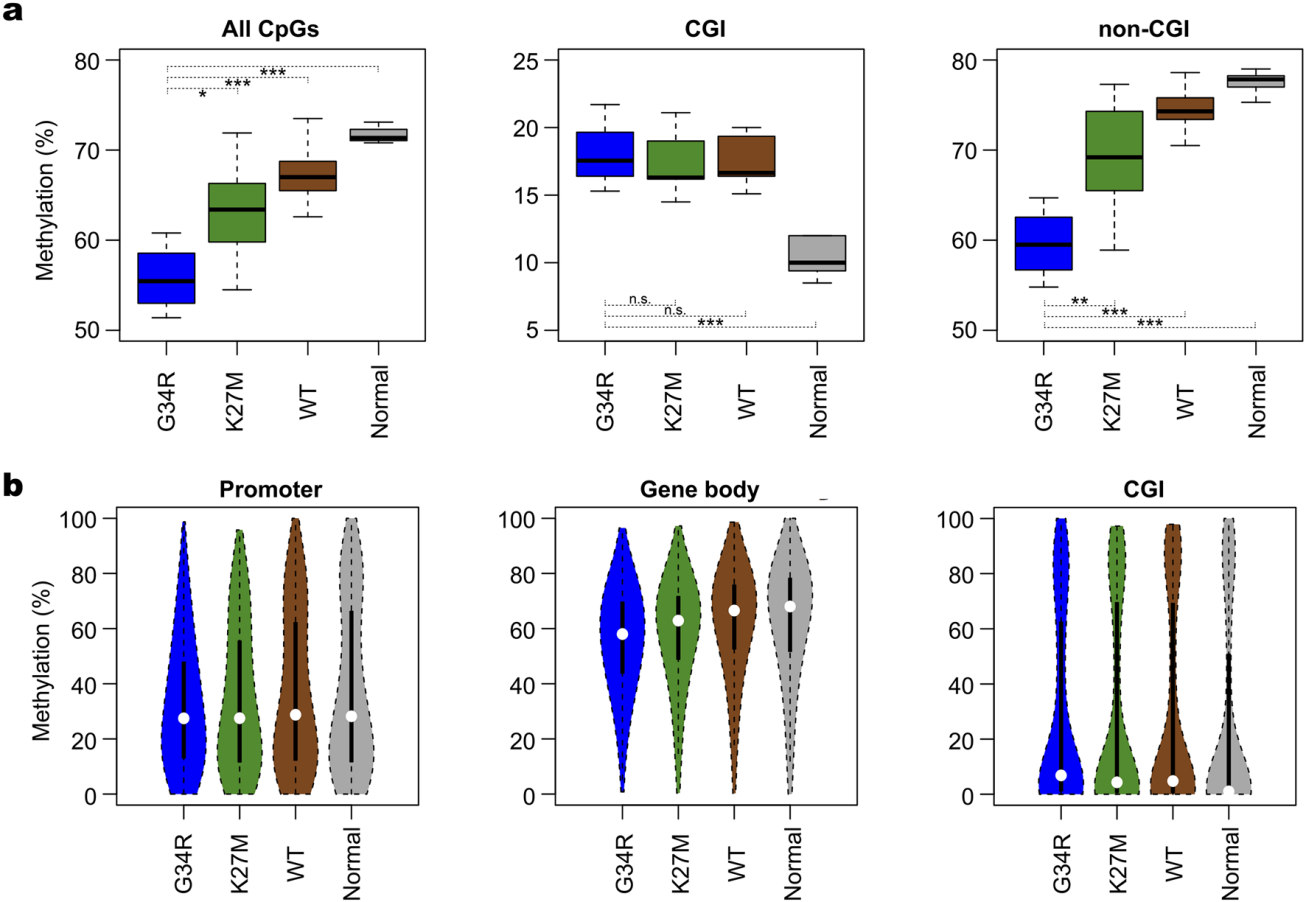
Global methylation levels of gliomas and normal brain tissue. (**a**) Box plots of mean CpG methylation levels. Data on all CpGs, those in CGIs, and those in non-CGI regions are shown for the three glioma subgroups and the normal brain tissue (*p < 0.05; **p < 0.01; ***p < 0.001; n.s., not significant). G34R, N = 4; K27M, N = 13; WT, N = 8; Normal, N = 8. (**b**) Violin plots of CpG methylation levels in promoters, gene bodies, and CGIs. Mean methylation levels are calculated for non-overlapping 1-Mb sliding windows.

We used the PBAT method to conduct PCR-free WGBS of four, seven, and three cases of primary gliomas harboring G34R mutation (G34 subgroup), K27M mutation (K27 subgroup), and no mutation in the *H3F3A* gene (WT subgroup), respectively, as well as the G34V-mutated glioma cell line KNS-42 (Tables S1 and S2). Combining these WGBS datasets with those obtained from the ICGC database, we compared the mean CpG methylation levels among the three subgroups. G34 subgroup exhibited the lowest mean methylation level (55.8%), which was followed by those of K27 (63.6%) and WT (67.3%) subgroups (Fig. 1). This order was maintained in the promoters and gene bodies but not in CGIs; mean CGI methylation levels were comparable among the three subgroups (Fig. 1). All the three subgroups exhibited lower global and higher CGI methylation than the normal brain tissues.

To further evaluate the methylation pattern among the three subgroups, we divided the genome into non-overlapping 1-Mb sliding windows, calculated mean methylation levels of individual windows, and examined their distribution (Figure S1a). While the methylation means of the windows exhibited a unimodal distribution in each subgroup, the peak of G34 subgroup was relatively the most left-shifted (i.e., hypomethylated) and broadest among the three subgroups. When normalized to each genome-wide methylation mean, the G34 subgroup showed a wider distribution in both directions (i.e., relative hypermethylation and hypomethylation) compared to the other two subgroups, indicating a larger variance in local methylation levels (Figure S1b).

We also analyzed partially methylated domains (PMDs), which are large genomic regions with intermediate methylation levels and often overlap with the lamina-associated domains (LADs)^26^. Almost all PMDs common to the four G34R-mutated gliomas overlapped with those identified in at least one case of the other two subgroups (Figure S2). Thus, there were likely very few, if any, qualitative differences of PMDs between G34 and the other two subgroups.

### Mitigation of telomere-proximal CGI hypermethylation in G34-mutated glioma

**Figure 2:**
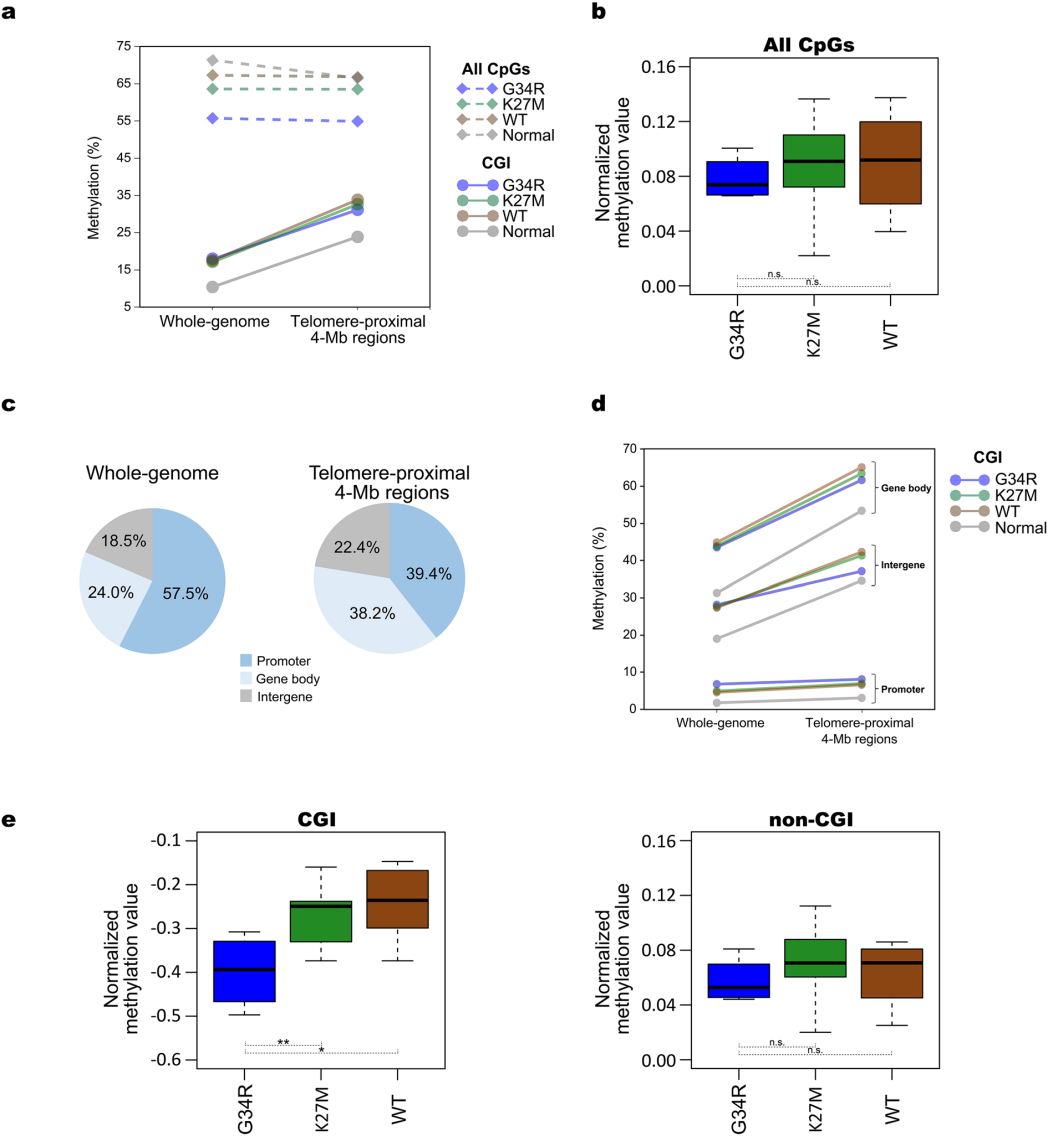
Methylation trends in telomere-proximal regions. (**a**) Mean methylation levels of whole-genome and telomere-proximal 4-Mb regions. Mean methylation levels are separately plotted for all CpG sites and those within CGIs. Only mean values of individual sample categories are indicated for clarity. G34R, N = 4; K27M, N = 13; WT, N = 8; Normal, N = 8. (**b**) Normalized mean methylation value of telomere-proximal 4-Mb regions. The methylation value was calculated using all CpG sites as follows. Mean methylation level of telomere-proximal 4-Mb regions of each sample category was first normalized by its whole-genome mean methylation level. The normalized value of each tumor subgroup was further normalized to that of the normal brain tissue. Log2-values are indicated. (**c**) Compositional difference of CGIs between whole-genome and telomere-proximal regions. CGIs are divided into those at promoters, gene bodies, and the other regions (intergenic regions). (**d**) Mean methylation levels of three distinct CGI classes in whole-genome and telomere-proximal 4-Mb regions. (**e**) Normalized mean methylation values of CGIs and non-CGI regions in telomere-proximal regions. Each methylation value was calculated as in (**b**).

A previous methylation array study reported “enhanced hypomethylation” at the chromosomal ends (i.e., telomere-proximal 4-Mb regions) as a characteristic of G34-mutated glioma^12^. However, current WGBS data indicated that mean methylation levels of these regions are comparable to the genome-wide mean in all the three subgroups (Fig. 2a, broken lines). In the normal brain tissues, methylation of these regions was lower than the genome-wide mean (Fig. 2a). Even after normalization to the normal brain tissue, telomere-proximal 4-Mb regions failed to show a significant difference among the three subgroups in contrast to the previous study^12^ (Fig. 2b).

To reconcile this apparent discrepancy, we separately analyzed CGIs and non-CGI portions of the genome. Notably, the mean CGI methylation level in telomere-proximal 4-Mb regions was higher than the genome-wide mean in both normal and tumor samples (Fig. 2a, solid lines). To address the reason for this elevated methylation, we classified CGIs into those at promoters, gene bodies, and intergenic regions. Intriguingly, CGIs at gene bodies and promoters were over- and underrepresented, respectively, in telomere-proximal 4-Mb regions, compared to the genome-wide mean (Fig. 2c). Gene body CGIs showed the highest mean methylation level, which was further elevated near the telomeres (Fig. 2d). By contrast, promoter CGIs showed the lowest mean methylation level with little, if any, sign of the telomere-associated elevation (Fig. 2d). These differences likely contributed to the observed elevation of CGI methylation levels at the chromosomal ends.

We next compared the extent of elevation in telomere-proximal CGI methylation among the three subgroups. Compared to K27 and WT subgroups, G34 subgroup showed a sign of mitigation in the elevation of methylation (Fig. 2a, solid lines), which was most evident in the intergenic CGIs (Fig. 2d). Indeed, when normalized to the normal brain tissue, G34 subgroup showed a significantly lower normalized methylation value than the other two subgroups (Fig. 2e, left). Importantly, however, non-CGI portions of telomere-proximal regions failed to show any significant difference in the normalized methylation values among the three subgroups (Fig. 2e, right). In the unbiased sampling by WGBS, the number of interrogated CpG sites from non-CGI portions overwhelms those from CGIs. Hence, current WGBS data likely masked the methylation changes at CGIs by those at non-CGI portions, leading to the apparently comparable methylation values among the three subgroups (Fig. 2b).

Taken together, while all gliomas exhibited the elevation of CGI methylation level in telomere-proximal regions, its extent was less prominent in the G34 subgroup compared to the other two subgroups.

### Methylation changes specific to G34-mutated gliomas

To identify the most characteristic methylomic changes, we aimed to identify differentially methylated regions (DMRs) between G34 and the other two subgroups (i.e., G34 subgroup-specific DMRs). For this purpose, we first identified DMRs between the G34 and K27 subgroups, between the G34 and WT subgroups, and between the G34 subgroup and the normal brain tissue. Next, we determined the intersection of these three DMR sets to define 687 G34 subgroup-specific hyper-DMRs (G34-hyper-DMRs; 0.2 Mb in total) and 2,064 G34 subgroup-specific hypo-DMRs (G34-hypo-DMRs; 1 Mb in total) (Figure S3a; Table S3). Similarly, we identified K27-specific DMRs, WT-specific DMRs, and DMRs common to all the three subgroups (Figures S3b–S3f).

**Figure 3:**
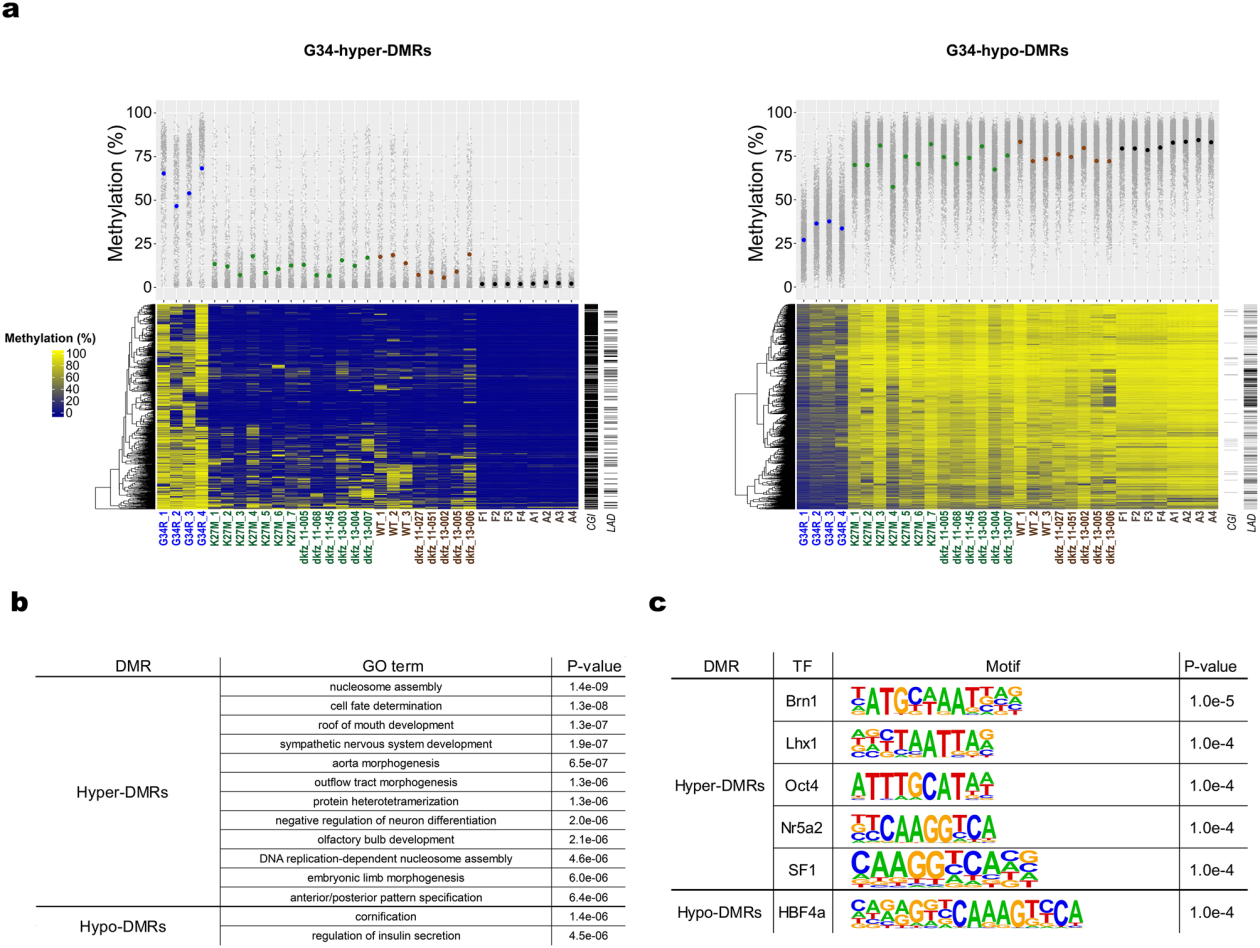
Hypermethylated and hypomethylated regions specific to the G34 subgroup. (**a**) G34-hyper-DMRs and G34-hypo-DMRs. Raw methylation levels are used for hierarchical clustering of DMRs. Rows and columns of the heatmap indicate DMRs and samples, respectively. Ladders on the right side of each panel indicate CGIs and LADs. G34R, N = 4; K27M, N = 13; WT, N = 8; Normal, N = 8. (**b**) GO enrichment analysis of DMRs. See Table S3 for details. (**c**) Transcription factor-binding motifs enriched in DMRs.

G34-hyper-DMRs and G34-hypo-DMRs were enriched for CGIs and LADs, respectively (Fig. 3a). Genes proximal to G34-hyper-DMRs were enriched for those associated with the GO term “nucleosome assembly”, including canonical histone genes, especially, those in the *HIST1* cluster (Fig. 3b; Table S3). They were also enriched for genes involved in neural differentiation, including those encoding oligodendrocyte-specific transcription factors OLIG1 and OLIG2, consistent with a previous study^12^. The binding motif of BRN1/POU3F3, a POU family transcription factor involved in neural development, was overrepresented in G34-hyper-DMRs (Fig. 3c). On the other hand, G34-hypo-DMRs failed to enrich notable proximal genes and transcription factor-binding motifs (Fig. 3b,c; Table S3).

As an alternative approach to identifying G34 subgroup-specific alterations, we applied independent component analysis (ICA) to the WGBS data. ICA is a method for blind source separation to decompose a data matrix into the product of a mixing matrix and independent components (ICs), the latter of which represent hidden source signals^27^. The elements of the mixing matrix can be used for hierarchical clustering of samples, based on the rationale that biologically similar samples should share a similar pattern in weighting to individual source signals. The mixing matrix also helps identify subgroup-specific ICs, which are weighted either positively or negatively in certain subgroups, but not in the others. Major contributors showing large, either positive or negative, loading factors (LFs) to such a subgroup-specific IC should serve as good methylation markers for the subgroup.

**Figure 4:**
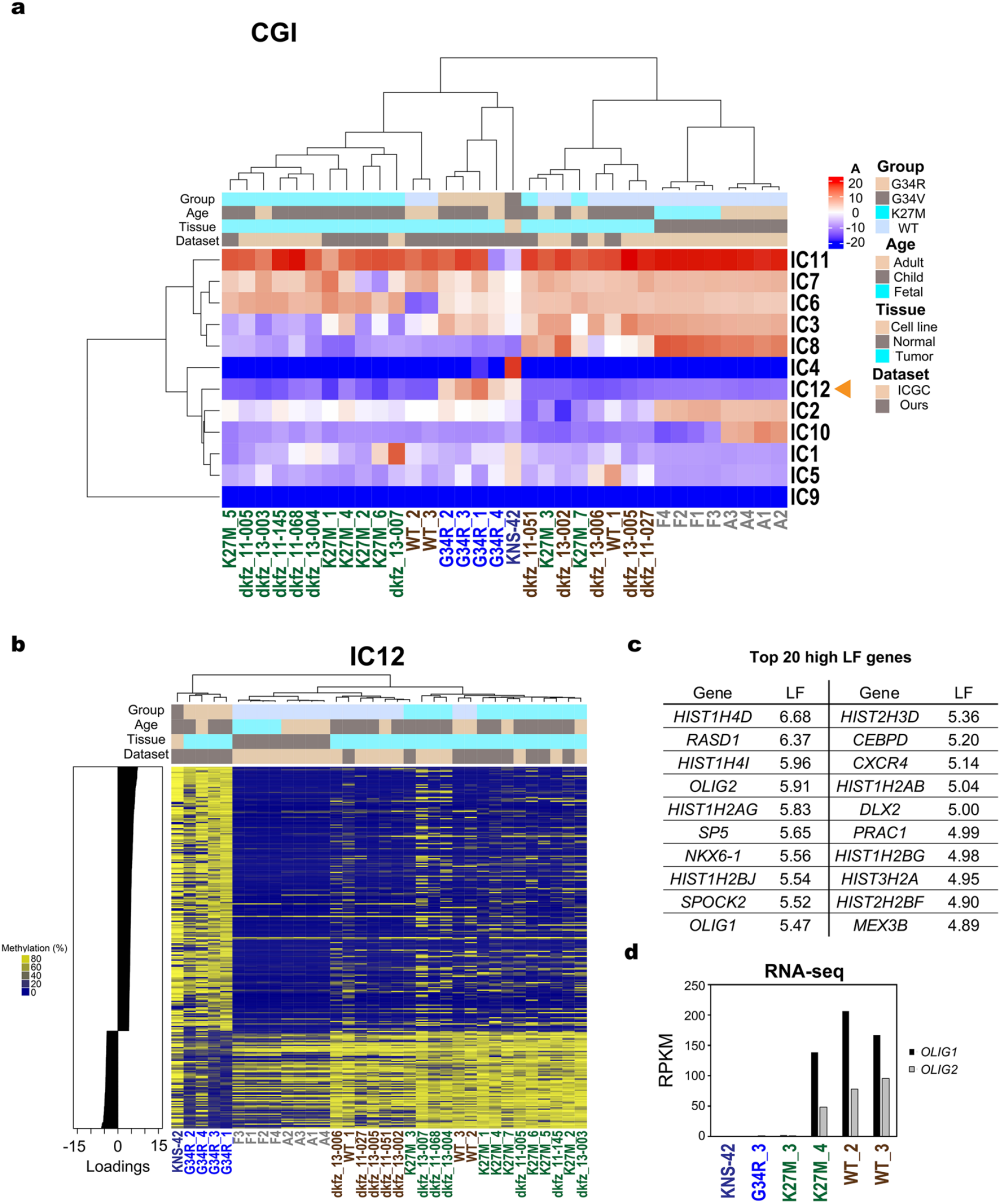
Independent component analysis (ICA). (**a**) Unsupervised hierarchical clustering of samples and ICs by mixing matrix coefficients. Results are shown for ICA of CGIs. Arrowhead indicates IC12 showing distinct weighting patterns between the G34 subgroup and the others. G34R/V, N = 5; K27M, N = 13; WT, N = 8; Normal, N = 8. (**b**) Unsupervised hierarchical clustering of samples by raw methylation levels of CGIs with high loading factors (LFs) for IC12 of ICA using CGIs. CGIs with LFs larger than 3 or smaller than − 3 are selected for the hierarchical clustering. (**c**) List of the top 20 genes with high LFs for G34 subgroup-specific IC of ICA using gene bodies. (**d**) Expression levels of *OLIG1* and *OLIG2*. RPKM values calculated from RNA-seq data are shown for the indicated samples.

We separately applied ICA to the methylation data for promoters, gene bodies, and CGIs (Figs. 4a and S4a). In hierarchical clustering based on the mixing matrix, all G34R-mutated gliomas and KNS-42 formed a monophyletic cluster excluding the others when CGIs and gene bodies were used in ICA (Figs. 4a and S4a). On the other hand, two K27M-mutated gliomas were co-clustered with the G34R-mutated gliomas in the ICA of promoters (Figure S4A). Note that IC12 (CGIs), IC3 (gene bodies), and IC3 (promoters) exhibited significantly different weighting patterns between G34 and the other two subgroups, thus serving as G34-specific ICs (Figs. 4b and S4).

Genes with large-positive LFs to these G34 subgroup-specific ICs were enriched for canonical histone genes in the *HIST1* cluster and the GO term “nucleosome assembly” (Fig. 4c; Table S4), consistent with the DMR analysis (Fig. 3; Table S3). The large positive LF genes also included *OLIG1* and *OLIG2*, silencing of which was indicated by RNA-seq in the G34 subgroup (Fig. 4d). On the other hand, genes with large negative LFs failed to enrich any notable GO terms (Table S4).

### G34 variant-specific methylomic signature shared between gliomas and bone tumors

Mutations affecting the G34 residue of histone H3.3 are prevalent in both gliomas and GCTBs. The G34W mutation occurs in most, if not all, GCTBs and a fraction of osteosarcomas. A study of GCTBs and osteosarcomas using methylation array attracted our attention, because it identified *HIST1H2BB* as the most differentially methylated gene between osteosarcomas with and without G34W mutation^15^ and because this gene was found within G34-hyper-DMRs and among the large-positive LF genes for G34 subgroup-specific ICs in our study (Tables S3 and S4). We thus wondered whether the G34W mutation in bone tumors induces methylomic changes resembling those induced by G34R mutation in gliomas.

**Figure 5:**
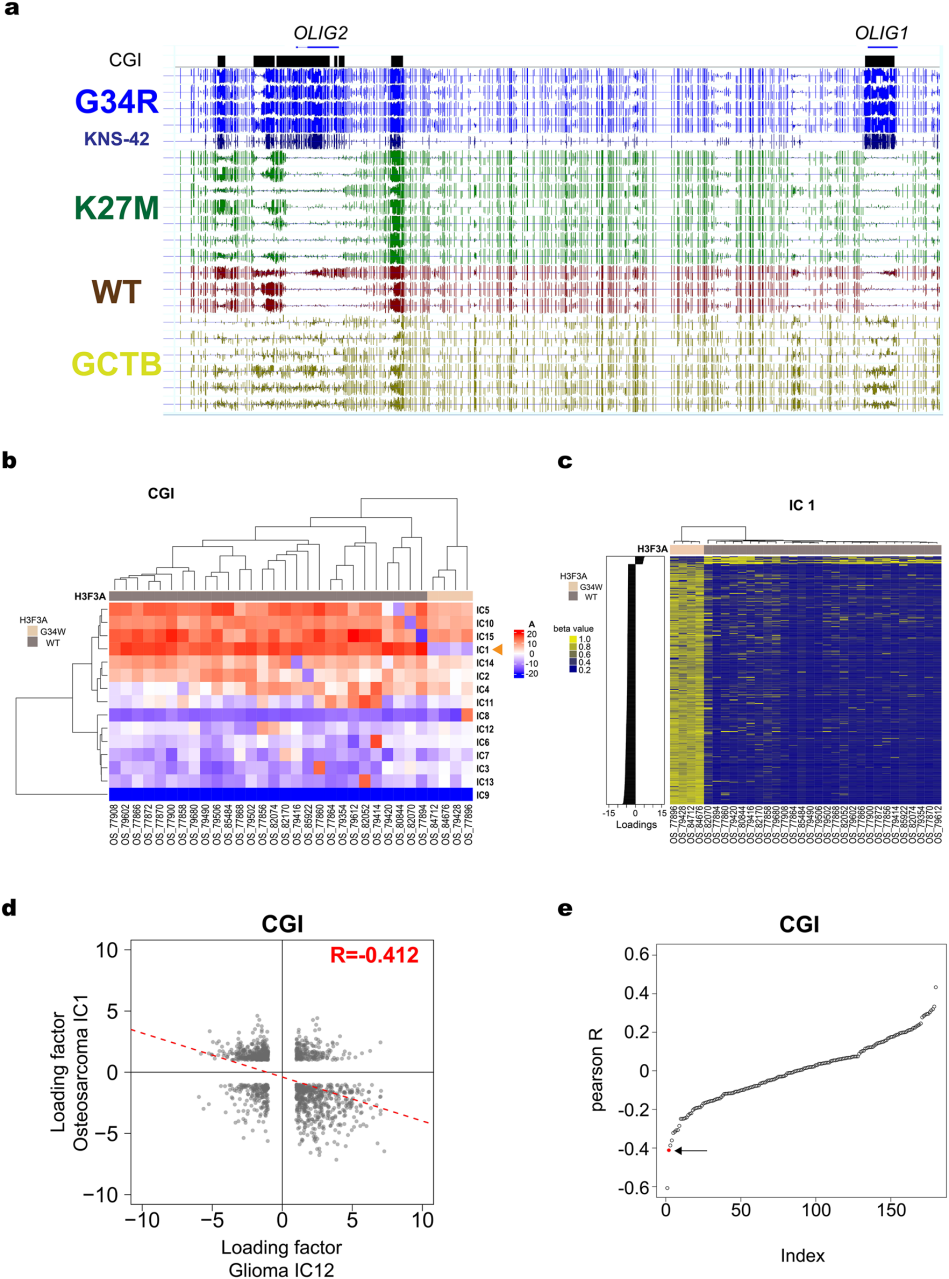
Methylomic signature shared by gliomas and bone tumors bearing G34 mutations. (**a**) Genome browser shot of *OLIG1/OLIG2* locus in gliomas and GCTBs. (**b**) Unsupervised hierarchical clustering of osteosarcoma samples and ICs by mixing matrix coefficients. Results are shown for ICA of CGIs based on the methylation array data (N = 32)^15^. Arrowhead indicates IC1, which shows distinct weighting patterns between tumors with and without G34W mutation. (**c**) Unsupervised hierarchical clustering of osteosarcoma samples by raw methylation levels of CGIs. Genes with LFs larger than 3 or smaller than − 3 are used for clustering. (**d**) Correlation plot of LFs between glioma IC12 and osteosarcoma IC1, both specific to G34 subgroup. ICA was performed using CGIs. (**e**) Pearson’s correlation coefficients of all possible pairs between ICs identified in ICA of glioma and osteosarcoma. ICA uses CGIs and the coefficients are sorted by their values. Red circle pointed by the arrow indicates the pair of G34-specific ICs (i.e., glioma IC12 and osteosarcoma IC1).

To directly address this possibility, we performed shallow WGBS on six GCTB cases, all of which were confirmed to harbor the G34W mutation^16^, and found that the *HIST1* cluster, *OLIG1*, and *OLIG2* exhibited modest levels of hypermethylation (Fig. 5a). Judging from the mutant allele frequency revealed by exome sequencing (0.09–0.24)^16^, we attributed the modest levels to the relatively low tumor cell content in these specimens. However, it remains possible that the G34W mutation was not related to the observed methylation changes because no data was available for GCTB cases lacking *H3F3A* mutations.

We thus applied ICA to the methylation array data on osteosarcomas including both tumors with and without G34W mutation^15^. Interestingly, ICA readily identified G34W-specific ICs. For instance, ICA of CGIs identified IC1 to be G34W-specific (Fig. 5b,c). Similarly, ICA of promoters and gene bodies identified IC9 and IC12, respectively, as G34W-specific ICs, major contributors to which were notably enriched for genes associated with the GO term “nucleosome assembly” or noncanonical histone genes in the *HIST1* cluster (Figure S5a, Table S5). In addition, *OLIG1* and *OLIG2* showed large negative LFs to G34W-specific IC12 (*OLIG1*−5.71, *OLIG2*−4.28, Table S5).

We reasoned that if G34 mutations have similar methylome-wide effects in gliomas and osteosarcomas, G34 mutation-specific ICs identified in these two tumors should show an overall similarity. Indeed, LFs of highly contributing CGIs in glioma IC12 and osteosarcoma IC1 showed a good correlation (Fig. 5d). Note that the correlation was negative, because positive and negative LFs corresponded to hypermethylation in the glioma and osteosarcoma ICA, respectively (Figs. 4b, 5c). To evaluate the correlation further, we calculated Pearson’s correlation coefficients (PCCs) in all possible combinations between glioma and osteosarcoma ICs identified in ICA of CGIs. Notably, G34 mutation-specific ICs of glioma and osteosarcoma showed the mutual top-hit relation: glioma IC12 showed the negatively largest PCC with IC1 among 15 osteosarcoma ICs, whereas osteosarcoma IC1 showed the negatively largest PCC with IC12 among 12 glioma ICs. The pair of G34 mutation-specific ICs was in those displaying the highest PCCs among the 180 (= 12 × 15) possible combinations (Fig. 5e). We confirmed similar findings using the methylation array data of G34-mutated gliomas^12^ instead of the WGBS data (Figures S5b and S5c).

Taken together, these results suggested that G34 mutations exert a characteristic methylome-wide effect regardless of the tumor tissue of origin, presumably through a common underlying mechanism.

### Possible role of G34-substituted histone H3.3 in CGI methylation

**Figure 6:**
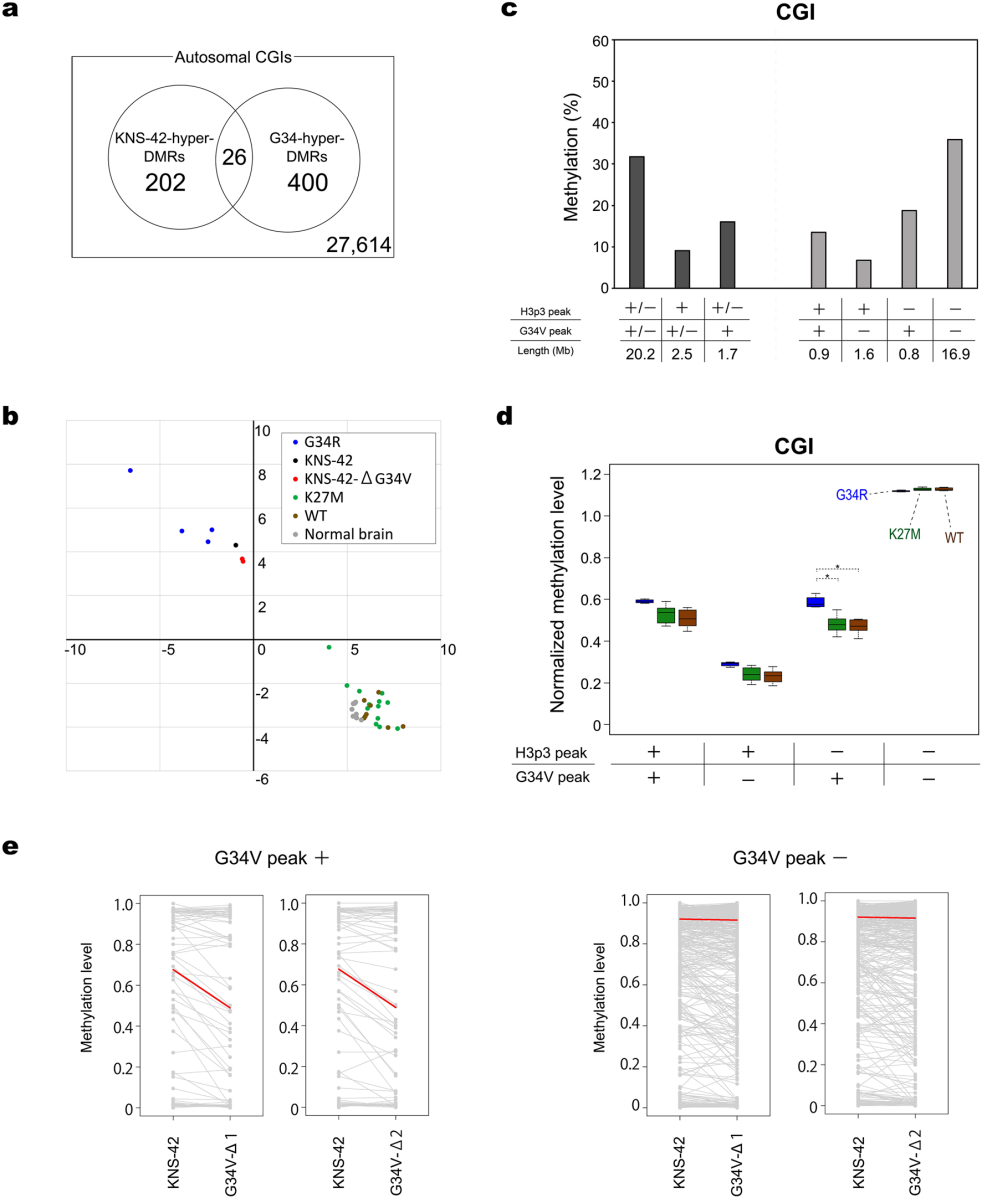
Effect of G34-substituted histone H3.3 on CGI hypermethylation. (**a**) Overlap between CGIs in G34-hyper-DMRs and those in KNS-42-hyper-DMRs. *p *value = 2.4 × 10^−15^; odds ratio = 8.7 (Fisher’s exact test). (**b**) Effect of G34V-allele disruption on G34-specific methylomic signature in KNS-42 cells. ICA including the two disruptants identified two IC’s to be G34-specific, one from ICA using CGIs and the other from ICA using promoters (Figure S6d). For each sample, weightings to the former and latter ICs are plotted to horizontal and vertical axes, respectively. Note that both disruptants depart from their parental KNS-42 cells toward the other 13 samples without G34 mutations, or in the opposite direction to four G34R gliomas. (**c**) Methylation levels of CGIs with differential histone H3.3 deposition patterns in KNS-42 cells. CGIs are classified into four groups based on the presence (+) or absence (−) of overlapping H3p3 and G34V peaks in KNS-42 cells. Each bar indicates mean methylation level of each CGI group. Length indicates the total length of genomic regions exhibiting the indicated ChIP-seq peak patterns. (**d**) Normalized methylation levels of the four CGI groups in clinical glioma samples. CGIs are classified as in **c**, and their methylation levels in glioma subgroups are displayed by box plots. Methylation levels are normalized to the genome-wide mean of individual tumor samples. G34, N = 4; K27M, N = 13; WT, N = 8. (**e**) Effect of G34V-allele disruption on G34-hyper-DMRs. Ladder charts are shown for methylation levels of CGIs in G34-hyper-DMRs in KNS-42 and each disruptant. CGIs in G34-hyper-DMRs are divided into those overlapping with G34V peaks (N = 92) and those without them (N = 334). Red lines indicate the median methylation change.

As an approach to address the mechanism underlying the G34-specific methylomic signature, we examined the effects of CRISPR/Cas9-mediated disruption of G34V-allele in KNS-42 cells. We selected two disruptants (Figures S6a–S6c) and identified DMRs between them and their parental KNS-42 cells by WGBS (Table S6). Although the overall effects of G34V-allele disruption were rather limited, CGIs in KNS-42-hyper-DMRs (i.e., genomic regions demethylated in the disruptants compared to KNS-42 cells) showed a significant overlap with CGIs in G34-hyper-DMRs (Fig. 6a; *p* = 2.4 × 10^−15^, odds ratio = 8.7). In ICA, the two disruptants had lower weightings on G34-specific ICs than their parental KNS-42 cells (Figs. 6b and S6d). It thus seemed that the contribution of G34-specific methylomic signature was diminished in the absence of G34V-substituted histone H3.3. These results suggested that G34V mutation contributes, at least, partly to maintain the G34-specific methylome signature.

We also performed ChIP-seq using two monoclonal antibodies, one specific to histone H3.3 and the other specific to histone H3.3-G34V. We hereinafter refer to ChIP-seq peaks obtained by the former and latter antibodies as H3p3 and G34V peaks, respectively. Both H3p3 and G34V peaks were enriched in CGIs and promoters in KNS-42 cells, sharing a largely similar distribution pattern (Figures S7a and S7b). We examined the overlap between ChIP-seq peaks and G34-hyper/hypo-DMRs. G34V peaks overlapped with 13% and 0.9% of G34-hyper-DMRs and G34-hypo-DMRs, respectively (Figure S7c). Note that G34-hyper-DMRs were enriched for CGIs (Fig. 3a; *p* < 2.2 × 10^−16^, odds ratio = 97.0) and that CGIs were enriched for H3p3 and G34V peaks (Figure S7a, *p* < 2.2 × 10^−16^; odds ratio = 7.72 for H3p3, 3.61 for G34V).

To determine the relationship between histone H3.3 deposition and CGI methylation, we first classified CGIs into four distinct groups based on the presence and absence of overlapping ChIP-seq peaks in KNS-42 cells and then compared methylation levels among the four groups. CGIs with histone H3.3 were less methylated than those without it, irrespective of whether the 34th residue was Gly or Val (Fig. 6c). Among histone H3.3-bearing CGIs, those with G34V peaks showed higher methylation levels than those without them (Fig. 6c). A similar trend was observed in clinical tumor samples, even in those without G34R mutation (Fig. 6d). It was thus possible that these CGIs were intrinsically prone to hypermethylation and that the G34 variants were preferentially incorporated to and/or stably retained in the hypermethylated nucleosomes. Intriguingly, however, the G34 subgroup exhibited a larger extent of hypermethylation than the other two subgroups (Fig. 6d). These results suggest an involvement of G34R variants in further elevating, or counteracting to decrease, CGI methylation levels. In this context, it is intriguing to note that CGIs in G34-hyper-DMRs with G34V peaks, but not those without them, were efficiently demethylated upon G34V-allele disruption in KNS-42 cells (Fig. 6e). Collectively, these results suggested an involvement of G34-substituted histone H3.3 in hypermethylation of a subset of CGIs.

## Discussion

We used the enhanced PBAT protocol^24^ to generate WGBS data on 14 gliomas, including the first four WGBS data on cases with histone H3.3-G34R. These data not only consolidated the findings in a previous study using the methylation array^12^ but extend the characteristics of G34-mutated gliomas, including a specific signature shared by G34-mutated bone tumors.

The previous study reported that G34-mutated gliomas exhibit two features, namely, global CpG hypomethylation and enhanced hypomethylation at the chromosomal ends^12^. In contrast to global hypomethylation (Fig. 1), enhanced hypomethylation at the chromosomal ends was not readily apparent in the WGBS data (Fig. 2). We demonstrated that the enhanced hypomethylation reported previously was based on the less prominent elevation of CGI methylation levels in telomere-proximal 4-Mb regions in the G34 subgroup compared to that in the other two subgroups (Fig. 2). It remains to be seen in future studies how G34 mutations, or coincident loss of ATRX, mitigates the elevation of CGI methylation levels in the telomere-proximal regions, a common feature shared between normal brain tissue and glioma.

We also identified methylomic changes specific to G34 subgroup using both conventional DMR search and ICA (Figs. 3, 4). We confirmed the previously reported G34 subgroup-specific hypermethylation and downregulation of *OLIG1* and *OLIG2*^12^. The requirement of *OLIG2* in gliomagenesis has been shown to be context-dependent^28^ and the pathogenic significance of its suppression in G34 subgroup remains an open question. We also found an enrichment of the binding motif of BRN1/POU3F3 in G34-hyper-DMRs. Hypermethylation around these sites may prevent BRN1/POU3F3 from binding to its target sites, thereby exerting an effect similar to its downregulation. In this context, it is interesting to note that the overexpression of *Linc-POU3F3*, a non-coding RNA that suppresses the expression of *POU3F3* mRNA, promotes cell viability and proliferation of glioma cells^29^. In addition, we revealed coordinated hypermethylation of canonical histone genes in the *HIST1* cluster, pathophysiological significance of which remains elusive. Since histone genes form a specific subnuclear structure called the histone locus body^30^, coordinated methylation changes may reflect alterations of the structure. A recent study on hypermethylation of histone-related genes in lung cancer proposed *HIST1H4F* hypermethylation as a potential pan-cancer biomarker, based on the analysis of 17 tumor types, not including glioma, from the TCGA database^31^. Our data, however, showed that hypermethylation of *HIST1H4F* occurs in G34 subgroup but not in the others. It is also intriguing to note that although the G34 subgroup seemed to show more unified methylomic changes than the K27 subgroup, tumor phenotypes such as the MRI findings and pathological diagnosis were more variable in the G34 subgroup than in the K27 subgroup^32,33^.

Furthermore, we applied ICA to the methylation array data on osteosarcomas to identify a G34W-specific signature and revealed its similarity to the G34-specific signature of gliomas (Fig. 5). Although GCTB seemed to share the signature, its clear identification was hampered by the unavailability of rare GCTB cases lacking G34 mutation and by the low tumor cell content in the samples. The latter issue may be overcome by single-cell methylome sequencing in future. In anyway, these results suggest that G34-mutated histone H3.3 exerts a characteristic methylomic effect regardless of the tumor tissue of origin, presumably through a common mechanism. Hence G34-specific DMRs identified in this study would provide good reporters to experimentally pursue the mechanism. These results also indicate a potential of ICA to uncover hidden methylomic signals.

How do G34 variants of histone H3.3 induce epigenomic changes including those of DNA methylation? Previous studies reported that G34R/V and G34W/L mutations locally affect di-/tri-methylation of K36 of histone H3 (H3K36me2/3) in the same nucleosome particle, when overexpressed in 293 T and HeLa cells, respectively^19,34^. Indeed, these substitutions were shown to inhibit SETD2, which is responsible for H3K36me2/3, in vitro^35^. On the other hand, a study using mouse embryonic stem cells showed that a knocked-in G34R allele induces widespread changes in H3K9me3 and H3K36me3 through inhibition of the KDM4 family of K9/K36 demethylases^36^. Although the effects of G34 mutations on H3K36me2/3 remain controversial, the well-established link between H3K36me3 and DNA methylation likely plays a role in methylomic alterations.

In this study, we used KNS-42 cells to examine the effects of G34V-allele disruption on the methylome and to reveal genomic distribution of G34V-substituted histone H3.3. These approaches are straightforward but have inevitably limitations. First, considering the context-dependent and feedback-rich nature of epigenetic regulation, we should note that simple disruption experiments allow us to examine only immediate effects on the maintenance, but not the generation, of methylome patterns. Molecular mechanisms for de novo formation of a methylome pattern can be dispensable for its maintenance, and the well-known recurrence of G34 mutations in distinct tumor types may be a reflection of developmental context-dependence of their epigenetic effects. Second, ChIP-seq results could be confounded if wild-type and mutant proteins are located at the same loci, because no antibody was available to distinguish wild-type histone H3.3 from G34-substituted proteins. Despite these limitations, our results collectively suggested a role for the deposition of G34-substituted histone H3.3 in elevating methylation levels of a subset of CGIs (Fig. 6). There should obviously be additional mechanisms that generate methylome patterns in genomic regions other than these CGIs. We expect that appropriate transgenic models for induced expression of G34 oncohistones and antibodies specific to wild-type histone H3.3 would help future studies to fully elucidate the mechanistic aspects and tumorigenic roles of the characteristic methylome patterns revealed in this study.

## Methods

### Glioma samples

From our previous comprehensive study analyzing *H3F3A* mutations in gliomas^25^, we included 11 *H3F3A*-mutated primary gliomas comprised of four G34R and seven K27M cases. As a control, we included three cases of age-matched glioblastomas, in which genetic analyses confirmed the absence of driver mutations, such as those in *IDH1/2*, *H3F3A*, and *BRAF* (V600E), in addition to 1p/19q codeletion^37^. These cases are summarized in Table S1. This investigation was approved by the Ethics Committee of Kyushu University.

### CRISPR/Cas9-mediated targeting of G34V allele in KNS-42 cells

The G34V-mutated glioma cell line KNS-42 was obtained from Japanese Collection of Research Bioresources Cell Bank. For CRISPR/Cas9-mediated disruption of G34V allele, a target DNA sequence (5′- TTC TTC ACC ACT CCA GTA GA-3′) was selected from the second exon of *H3F3A* with CHOPCHOP (New England Biolabs). A template oligonucleotide was designed with EnGen sgRNA Template Oligo Designer (New England Biolabs), synthesized by Eurofins genomics, and used to prepare the single guide RNA (sgRNA). A ribonucleoprotein complex formed between the sgRNA and EnGen Cas9 NLS, S. pyogenes (New England Biolabs) was electroporated into KNS-42 cells using NEON Transfection System (Thermo Fisher Scientific). Following the cloning by limiting dilution, the targeted region was amplified by PCR, cloned with Topo TA cloning kit (Thermo Fisher Scientific), and sequenced using BigDye Terminator v1.1 cycle sequencing kit and SeqStudio genetic analyzer (Thermo Fisher Scientific). Loss of G34V-substituted histone H3.3 was confirmed by Western blotting with anti-histone H3.3 G34V rabbit monoclonal antibody (RevMAb Biosciences, 31-1193-00).

### WGBS

We used an improved PBAT protocol based on a highly efficient method for single-stranded DNA ligation^24^ to prepare PCR-free WGBS libraries from 100 ng of genomic DNAs and subjected them to paired-end sequencing (150 nt × 2) using the Illumina HiSeq X Ten platform at Macrogen Japan Corp. (Kyoto, Japan). WGBS reads were processed as described previously^24^. The statistics are summarized in Table S2.

### Other methylome data

We obtained WGBS data on six and five high-grade gliomas bearing K27M and no mutation in *H3F3A* (EGAS00001000578), respectively, and eight normal cerebellum samples (EGAS00001000561) from the International Cancer Genome Consortium (ICGC). Methylation array data on GCTBs and osteosarcomas^15^ were obtained from the authors.

### Methylome bioinformatics

Autosomal CpG sites covered at least 10 times were used for downstream analyses. DMRs were identified using the program metilene with default parameters^38^. For clinical samples, DMRs were filtered to retain those containing at least 20 CpGs, with P-values less than 0.01 and with a methylation difference larger than 30%. For the comparison between KNS-42 with and without G34V allele, we used DMRs with P-values less than 10^−5^. ICA was performed using the fastICA package in R as described previously^39^. Outputs of ICA were used to generate Euclidian distance matrices for hierarchical clustering by Ward’s method. All heatmaps were generated using the ComplexHeatmap library^40^. Motif enrichment analysis was carried out using the findMotifsGenome.pl of HOMER^41^. All functional enrichment analyses with the Gene Ontology (GO) biological process terms were performed using the topGO package in R.

### RNA-seq

Total RNAs were prepared from frozen tissues of five glioma cases and KNS-42 cells using the RNeasy Mini Kit (Qiagen) with DNase treatment. RNA-seq libraries were prepared using the TruSeq stranded mRNA Kit (Illumina) and sequenced using NovaSeq6000 at Macrogen Japan Corp. RNA-seq reads were processed using the CLC Genomics Workbench (Qiagen).

### ChIP-seq

KNS-42 cells were fixed with 1% formaldehyde and incubated at room temperature for 15 min. The cells were collected with centrifugation and re-suspended in ChIP-lysis buffer (50 mM HEPES-KOH, pH7.5, 150 mM NaCl, 1 mM EDTA, 0.5% (v/v) sodium deoxycholate, 1% (v/v) NP-40, 0.1% (w/v) sodium dodecyl sulfate) containing 1 × protease inhibitor cocktail (Nacalai Tesque). The chromatin was sheared by sonication with Focused-ultrasonicator S220 (Covaris) using milliTUBE 1-ml AFA Fiber. For ChIP, anti-histone H3.3 rabbit monoclonal antibody (Abcam, ab176840) and anti-histone H3.3 G34V rabbit monoclonal antibody were used as primary antibodies with pre-washed Dynabeads Protein A (Dynal). The epitope of the former antibody lies between the 50th and C-terminal residues of human histone H3.3. The ChIP-ed DNA was purified using the DNeasy Blood and Tissue kit (Qiagen) with some modifications, and 10 ng of the DNA was used for library preparation with the SMARTer ThruPLEX DNA-seq Kit (Takara Bio) according to the manufacturer's instruction. Sequencing was performed using the HiSeq X Ten or NovaSeq 6000 at Macrogen Japan Corp. ChIP-seq reads were mapped to the reference human genome GRCh37 (hg19) and peak calling was performed using MACS2^42^.

## Supplementary information

Supplementary information.

Supplementary Table S3.

Supplementary Table S4.

Supplementary Table S5.

Supplementary Table S6.

## Data Availability

WGBS, RNA-seq, and ChIP-seq data have been deposited in the Japanese Genotype–phenotype Archive (JGA) under the accession number JGAS00000000197 and in the DDBJ Sequence Read Archive (DRA) under the accession numbers DRA010212 and DRA010335.
