# Supplementary material for: Base-resolution methylomes of gliomas bearing histone H3.3 mutations reveal a G34 mutant-specific signature shared with bone tumors
Source: Sci Rep. 2020 Sep 30;10:16162. doi: 10.1038/s41598-020-73116-x (PMC7527345; doi:10.1038/s41598-020-73116-x)
Supplement: Supplementary file 1 — Supplementary information. [file 41598_2020_73116_MOESM1_ESM.pdf]

Supplementary Information

Base-resolution methylomes of gliomas bearing histone H3.3 mutations reveal a G34 mutant-specific signature shared with bone tumors

Yuhei Sangatsuda[1,2], Fumihito Miura[1], Hiromitsu Araki[1], Masahiro Mizoguchi[2], Nobuhiro Hata[2], Daisuke Kuga[2], Ryusuke Hatae[2], Yojiro Akagi[2], Takeo Amemiya[2], Yutaka Fujioka[2], Yasuhito Arai[3], Akihiko Yoshida[4], Tatsuhiro Shibata[3], Koji Yoshimoto[2,5], Koji Iihara[2], and Takashi Ito[1,*]

1. Department of Biochemistry, Kyushu University Graduate School of Medical Sciences, Fukuoka, Japan
2. Department of Neurosurgery, Kyushu University Graduate School of Medical Sciences, Fukuoka, Japan
3. Division of Cancer Genomics, National Cancer Center Research Institute, Tokyo, Japan
4. Department of Diagnostic Pathology, National Cancer Center Hospital, Tokyo, Japan
5. Department of Neurosurgery, Graduate School of Medical and Dental Sciences, Kagoshima University, Kagoshima, Japan

*correspondence author
Takashi Ito,
Department of Biochemistry, Kyushu University Graduate School of Medical Sciences
3-1-3 Maidashi, Higashi-ku, Fukuoka 812-8582, Japan
Tel: +81-92-642-6095; Fax: +81-92-642-6203; E-mail: tito@med.kyushu-u.ac.jp

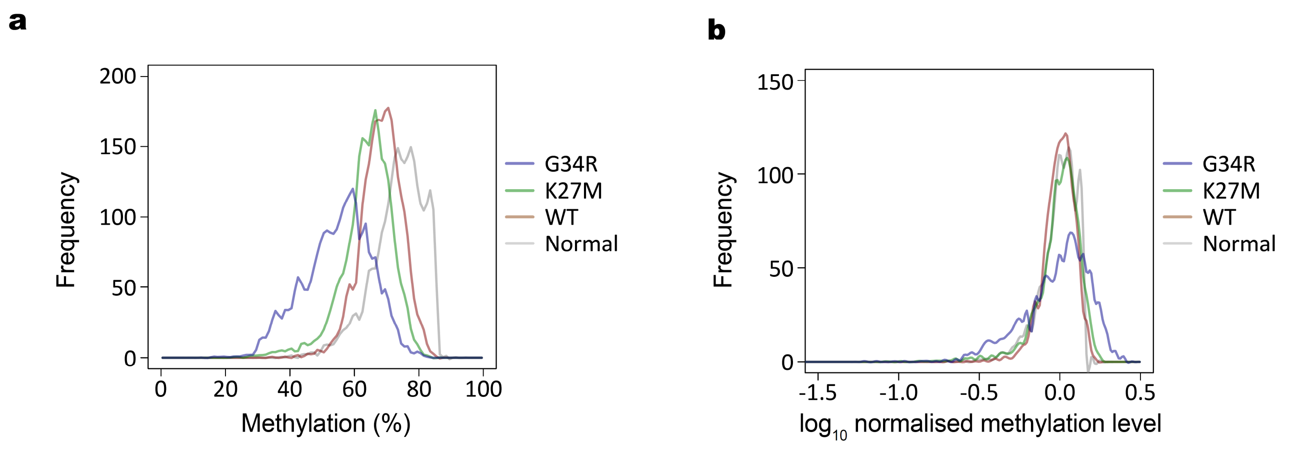

Figure S1. Regional variation of methylation levels

a. Methylation levels of non-overlapping 1-Mb sliding windows. Number of windows (vertical axis) are plotted against raw methylation levels (horizontal axis). G34R, N = 4; K27N, N = 13; WT, N = 8; Normal, N =8.

b. Normalized methylation levels of non-overlapping 1-Mb sliding windows. Number of windows (vertical axis) are plotted against log10-value of methylation levels normalized by genome-wide mean methylation levels.

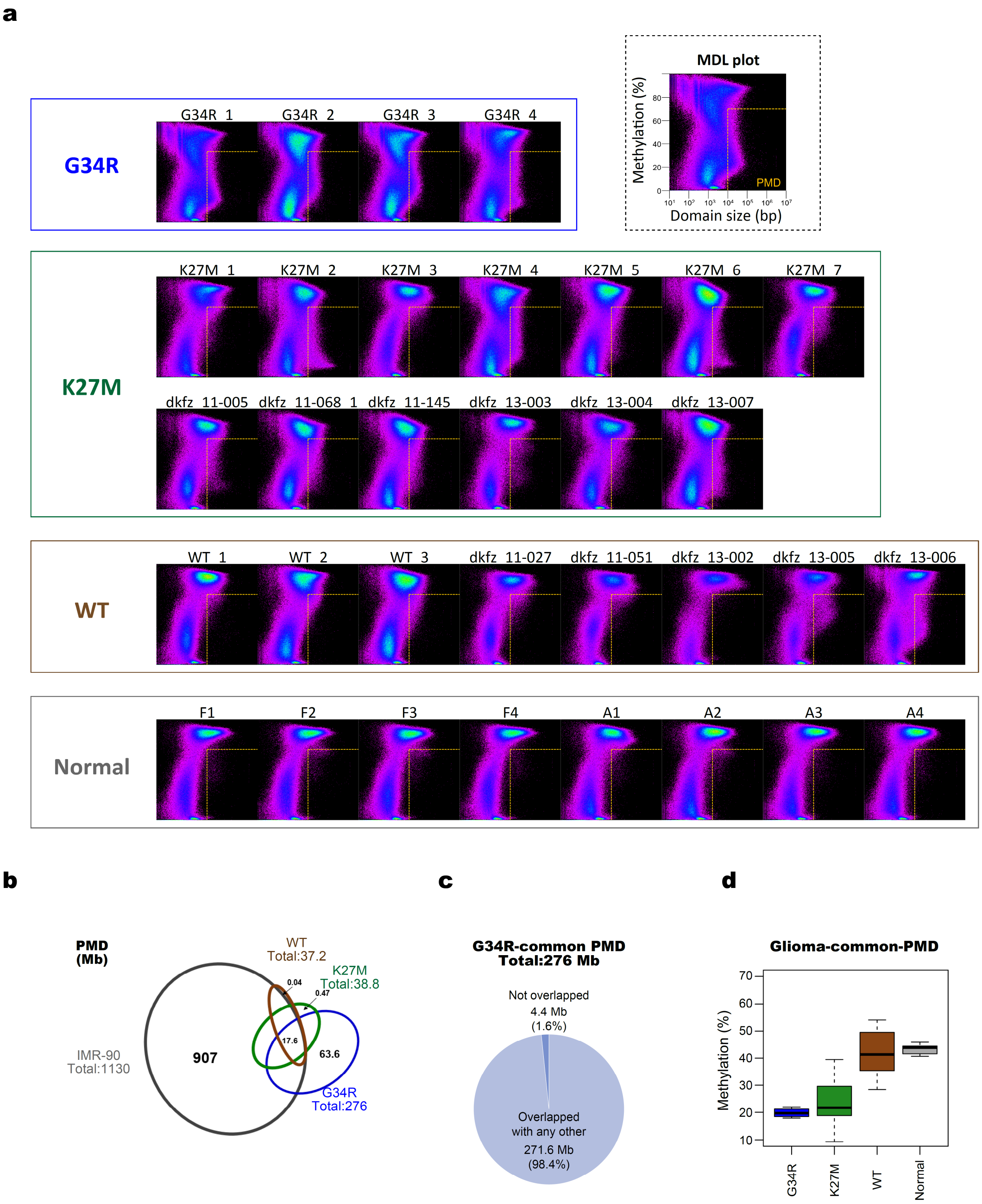

Figure S2. Partially methylated domains (PMDs)

a. Methylated Domain Landscape (MDL) plots. Methylated domains are defined using the changepoint detection algorithm[43]. The size and methylation level of each domain are plotted to the horizontal and vertical axes in MDL plot, respectively. PMDs defined as the original definition are demarcated by yellow dotted lines.

b. Overlap among common PMDs. Common PMDs are defined as those appeared in all samples of each glioma subgroup. Venn diagram indicates the overlap among the common PMDs of individual glioma subgroups and PMDs in fibroblast IMR-90 cells.

c. Overlap between G34R-common PMDs and those identified in the non-G34 cases. Almost all G34R-common PMDs (98.4%) overlapped with PMDs identified in at least one case of the non-G34 cases. Only 1.6% of G34R-common PMDs were specific to the G34 cases.

d. Methylation levels of glioma-common PMDs. The intersection of G34R-, K27M-, and WT-common PMDs are defined as the glioma-common PMDs (17.6 Mb). Box plots indicate mean methylation levels of the glioma-common PMD regions.

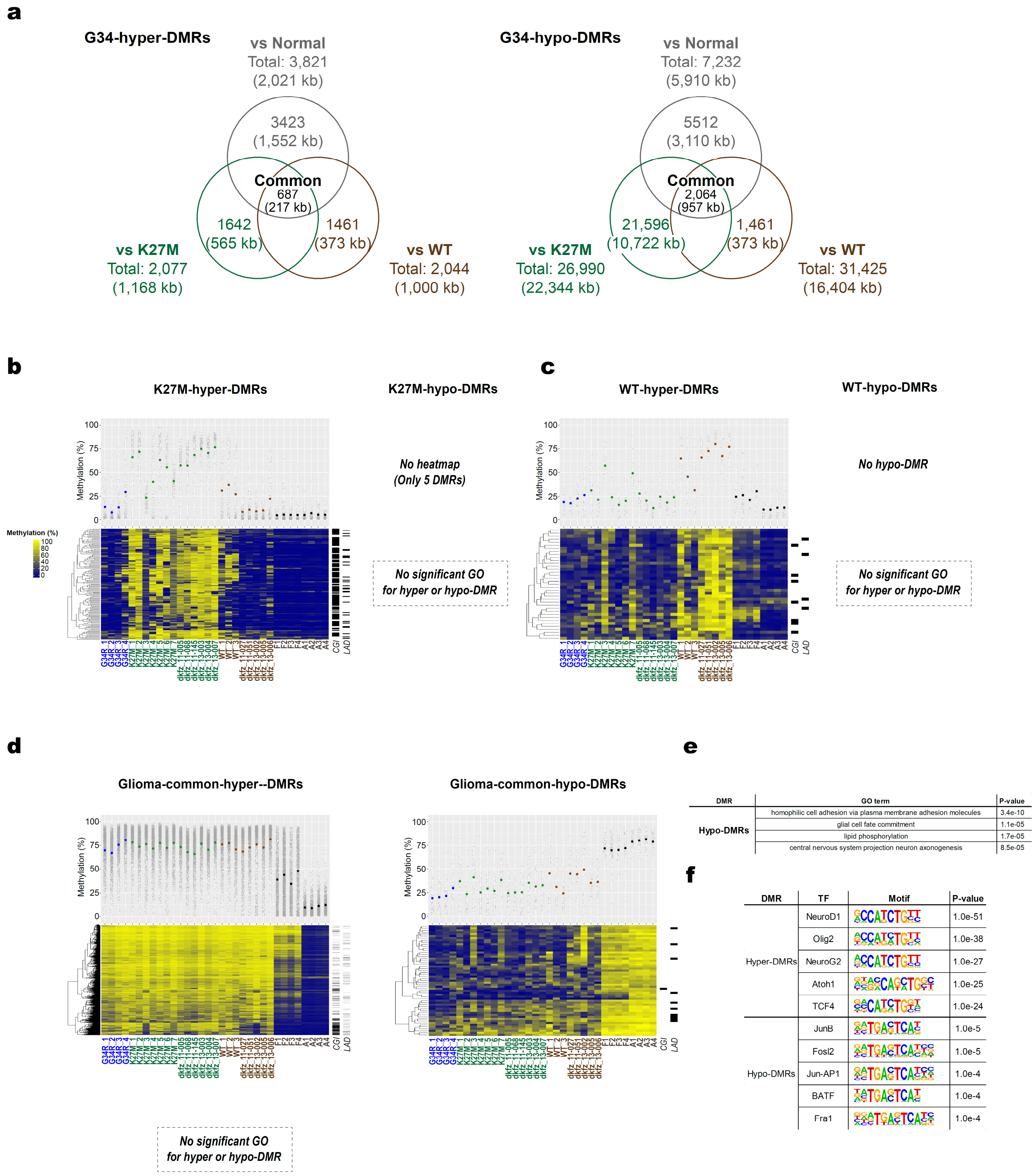

Figure S3. Differentially methylated regions (DMRs)

a. Venn diagrams for the definitions of G34-hyper-DMRs and G34-hypo-DMRs.

b. Hierarchical clustering of K27-hyper-DMRs by raw methylation levels.

c. Hierarchical clustering of WT-hyper-DMRs by raw methylation levels.

d. Hierarchical clustering of glioma-common hyper/hypo-DMRs by raw methylation levels.

e. GO terms enriched in genes proximal to glioma-common hypo-DMRs.

f. Transcription factor-binding motifs overrepresented in glioma-common DMRs.

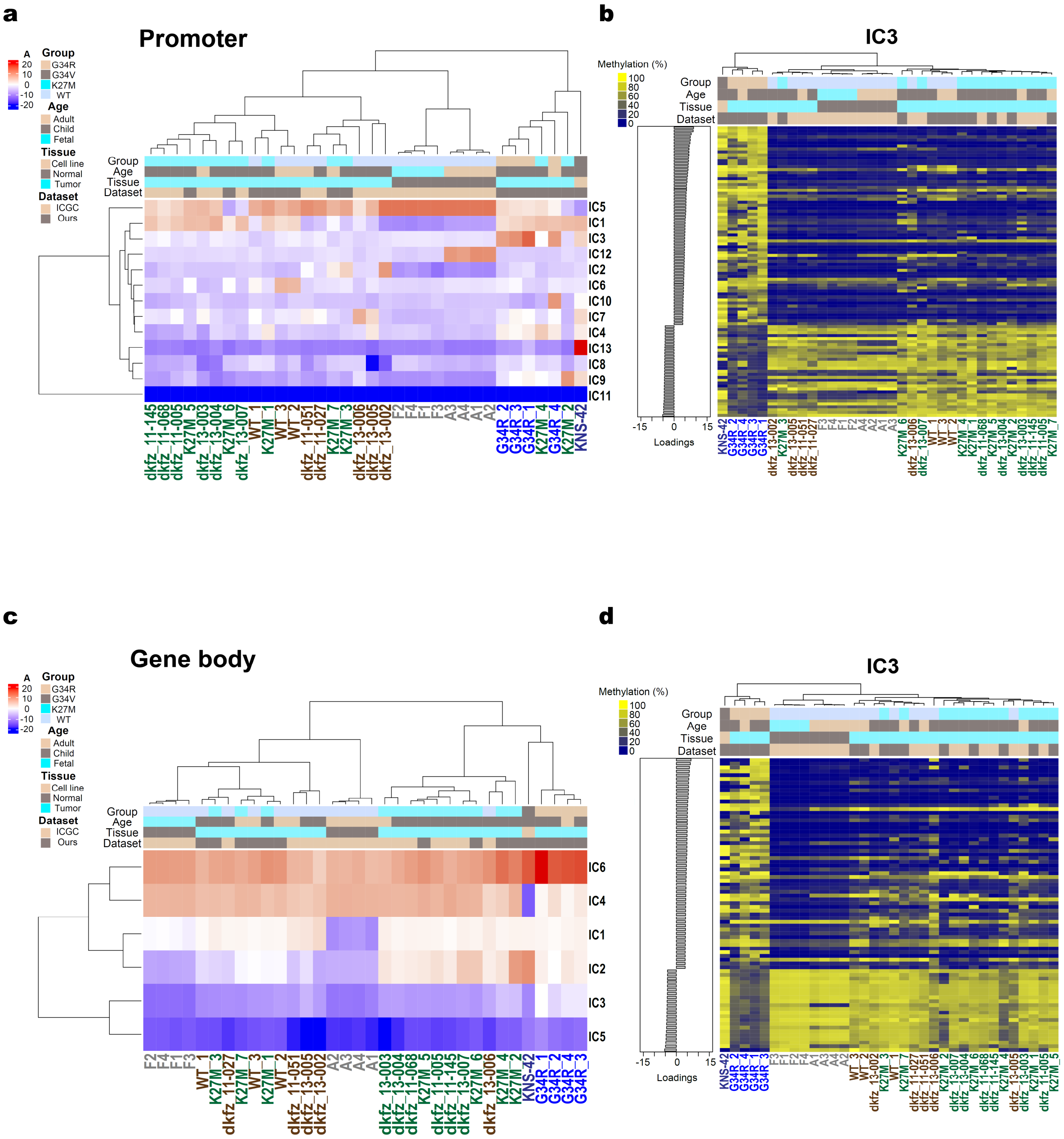

Figure S4. Independent component analysis (ICA) of glioma

- **a.** Unsupervised hierarchical clustering of samples and ICs by mixing matrix coefficients of ICA of promoters. Note that IC3 showing distinct weighting patterns between G34 subgroup and the others.
- **b.** Unsupervised hierarchical clustering of samples by raw methylation levels of promoters with large contribution to IC3 of ICA of promoters.
- **c.** Unsupervised hierarchical clustering of samples and ICs by mixing matrix coefficients of ICA of gene bodies. Note that IC3 showing differential weighting patterns between G34 subgroup and the others.
- **d.** Unsupervised hierarchical clustering of samples by raw methylation levels of gene bodies with large contribution to IC3 of ICA of gene bodies.

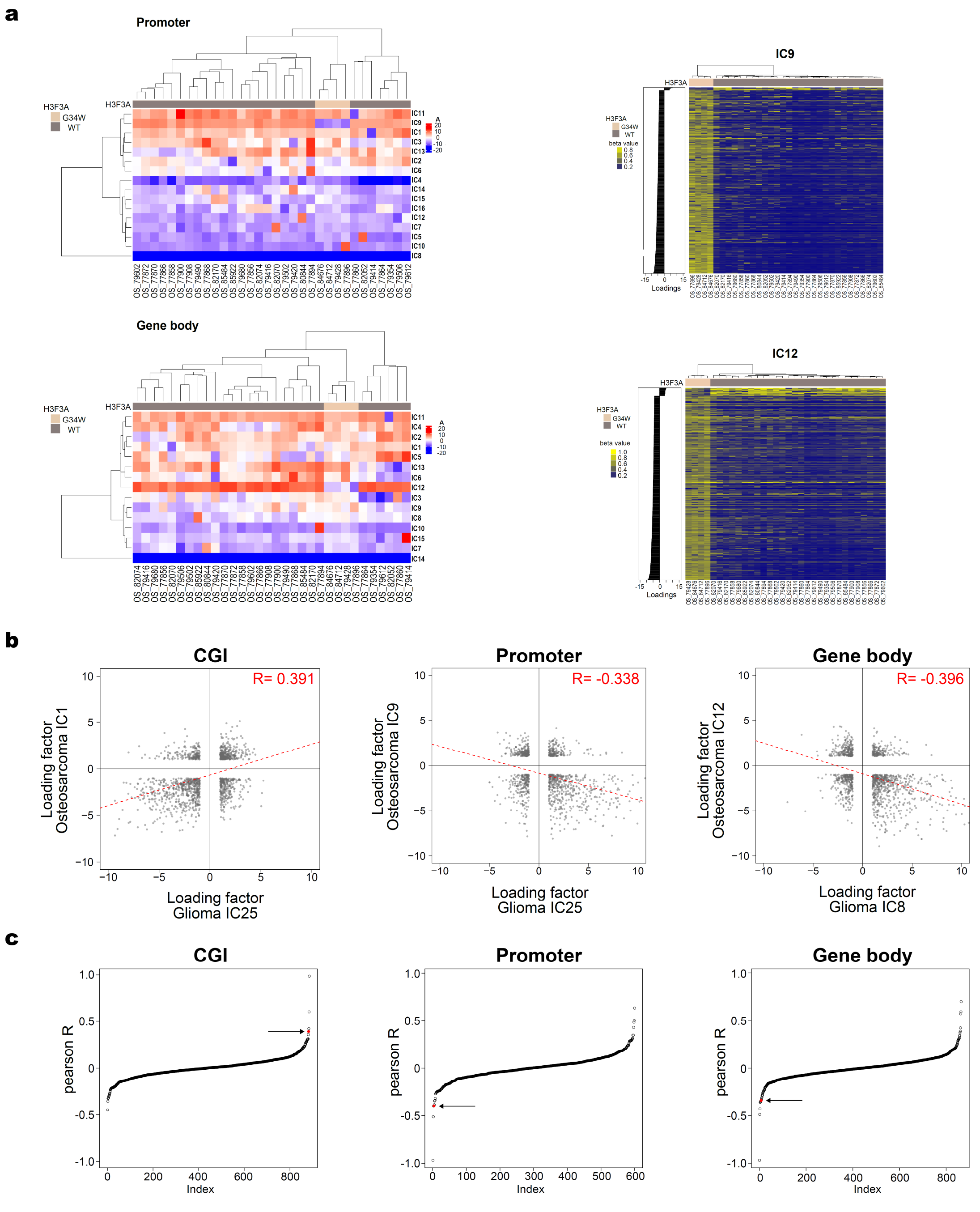

Figure S5. G34 mutation-specific ICs

a. Unsupervised hierarchical clustering of osteosarcomas. Heat maps are shown for two-way clustering of samples and ICs by mixing matrix coefficients (left panels) and for clustering of samples by raw methylation levels of major contributors (right). Results are shown for the ICA of promoters (top) and gene bodies (bottom) based on Infinium 450K methylation array data[15]. Note that IC9 (promoters) and IC12 (gene bodies) are G34W-speicific ICs.

b. Correlation plot of LFs between G34-specific ICs in glioma and osteosarcoma. In contrast to Figure 5d, glioma data is obtained by methylation array[12].

c. Distribution of PCCs between all possible combinations between ICs of glioma and osteosarcoma. In contrast to Figure 5e, glioma data is obtained by methylation array[12].

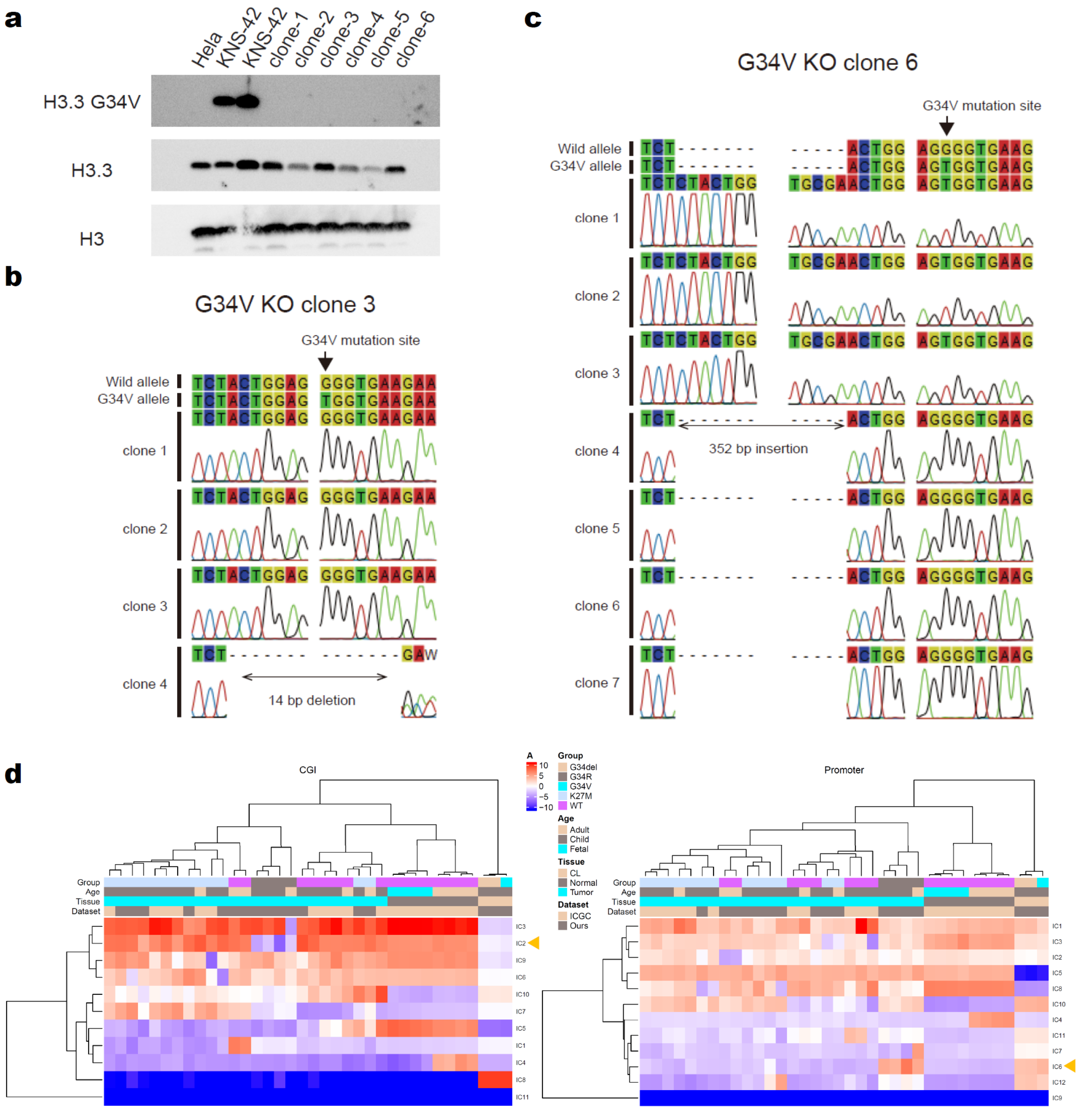

Figure S6. Generation of KNS-42 cells without histone H3.3-G34V

a. Western blotting. Six clones obtained by limiting dilution were subjected to immunoblotting for histone H3.3-G34V, histone H3.3, and histone H3. Hela and KNS-42 cells were included as negative and positive controls for histone H3.3-G34V, respectively. Clones 3 and 6 were used for subsequent analyses. Full-length blots/gels are presented in Figure S8.

b. *H3F3A* alleles in clone 3 (G34V-Δ2). It has an intact wild-type allele and a mutant allele disrupted by a 14-bp deletion.

c. *H3F3A* alleles in clone 6 (G34V-Δ1). It has an intact wild-type allele and a mutant allele disrupted by a 352-bp insertion.

d. ICA including the two disruptants. Left and right panels show ICA of CGIs and promoters, respectively. A tight cluster of KNS-42 and the two disruptants is formed as the rightmost of each panel. Arrowheads indicate G34-specific ICs (IC2 for ICA of CGIs, IC6 for ICA of promoters).

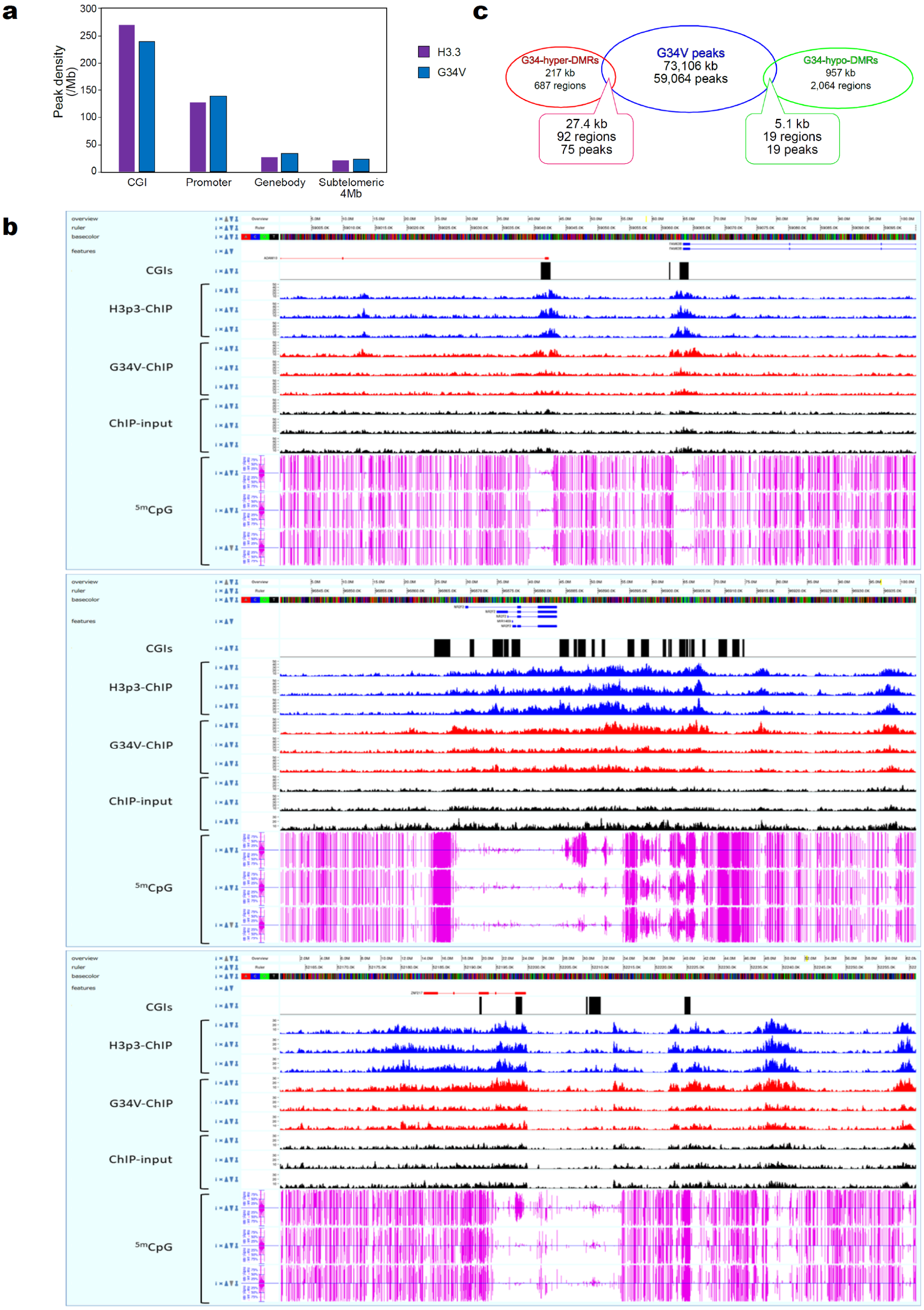

Figure S7. Deposition of histone H3.3 and CGI hypermethylation

a. Distribution of ChIP-seq peaks among genomic features. Peak densities are indicated for comparison.

b. Genome browser screenshots. In each triad, top track indicates KNS-42, whereas middle and bottom tracks indicate the two disruptants G34V-Δ1 and G34V-Δ2, respectively.

c. Overlap between G34-hyper/hypo-DMRs and ChIP-seq peaks for histone H3.3-G34V in KNS-42 cells.

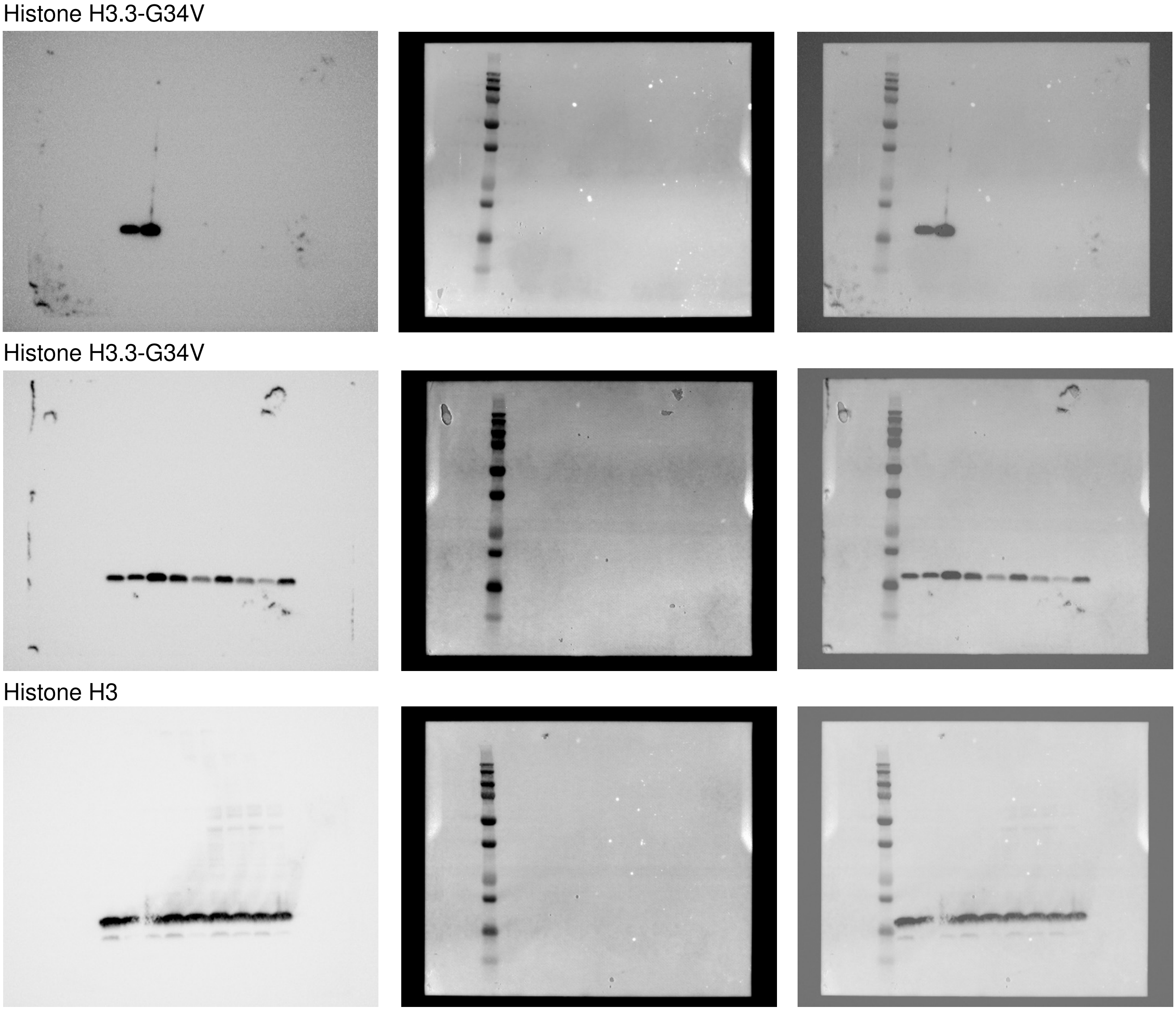

Figure S8. Full-length blots/gels
Full-length blots/gels are shown for the blots in Figure S6a. Top, middle, and bottom rows indicate histone H3.3-G34V, histone H3.3, and histone H3, respectively. Each row contains blot signals (left), size standards (center), and their merged image (right).

Table S1 Sample summary

ID	Age	Sex	Location	Histology	H3F3A	IDH1/IDH2	BRAF V600E	LOH				
								17p	1p	chr10	19q	1p/19q
G34R-1	10	F	Thalamus	Glioblastoma	G34R	WT/WT	WT	total	no	total	total	no
G34R-2	8	M	Temporal lobe	Glioblastoma	G34R	WT/WT	WT	total	no	no	total	no
G34R-3	11	M	Temporo-parietal lobe	Astroblastoma	G34R	WT/WT	WT	total	no	p partial	total	no
G34R-4	37	F	Temporal lobe	Glioblastoma	G34R	WT/WT	WT	no	no	total	total	no
K27M-1	8	M	Thalamus	Glioblastoma	K27M	WT/WT	WT	total	no	total	partial	no
K27M-2	15	F	Thalamus	Glioblastoma	K27M	WT/WT	WT	total	no	q partial	total	no
K27M-3	15	M	Pons-Cerebellum	Glioblastoma	K27M	WT/WT	WT	total	no	no	no	no
K27M-4	5	F	Thalamus	Glioblastoma	K27M	WT/WT	WT	total	no	total	partial	no
K27M-5	14	M	Thalamus	Glioblastoma	K27M	WT/WT	WT	total	no	no	no	no
K27M-6	6	M	Thalamus	Glioblastoma	K27M	WT/WT	WT	no	no	total	no	no
K27M-7	38	F	Thalamus	Glioblastoma	K27M	WT/WT	WT	total	no	total	no	no
WT-1	3	F	Intraventricule	Glioblastoma	WT	WT/WT	WT	no	no	no	no	no
WT-2	34	M	Bilateral frontal lobe	Glioblastoma	WT	WT/WT	WT	total	no	total	no	no
WT-3	26	F	Fronto-temporal lobe	Glioblastoma	WT	WT/WT	WT	total	no	p partial	no	no

Table S2 WGBS statistics

Sample type	ID	Total mapped nucleotides	Genome coverage	Bisulfite conversion rate (%)	Average CpG coverage	CpGs covered ≥5 times (%)	CpGs covered ≥10 times (%)	CpGs covered ≥20 times (%)	Mean methylation level (%)			
									all C	CpG	CHG	CHH
Primary MG	G34R_1	110,281,446,205	35.2	99.0	21.9	82.1	70.7	44.5	3.6	52.2	1.3	1.2
	G34R_2	93,589,333,674	29.8	99.0	17.5	80.5	66.4	35.0	3.7	56.0	1.3	1.2
	G34R_3	101,030,315,452	32.2	99.0	20.4	81.1	68.2	40.8	3.9	57.3	1.3	1.2
	G34R_4	127,869,444,528	40.8	99.1	25.5	80.9	70.2	47.9	4.1	61.8	1.3	1.2
	K27M_1	111,145,361,130	35.4	99.1	21.1	81.5	69.2	41.7	4.4	70.9	1.2	1.1
	K27M_2	68,016,854,629	21.7	99.0	12.7	72.6	51.8	18.2	3.7	54.8	1.3	1.2
	K27M_3	142,165,325,593	45.3	99.1	25.9	85.4	78.1	57.8	4.4	72.5	1.2	1.1
	K27M_4	83,878,628,912	26.7	99.0	16.5	76.1	59.3	28.7	4.4	67.8	1.4	1.3
	K27M_5	89,153,975,032	28.4	99.0	17.6	76.8	61.3	32.2	3.9	59.6	1.2	1.1
	K27M_6	105,267,499,634	33.6	99.0	21.5	78.3	65.8	41.3	4.0	60.6	1.3	1.2
	K27M_7	127,343,147,230	40.6	99.1	24.5	83.3	73.9	50.0	4.3	71.3	1.1	1.0
	WT_1	135,661,730,109	43.2	99.0	25.8	86.1	79.3	58.6	4.8	73.9	1.4	1.5
	WT_2	99,443,601,419	31.7	99.1	19.2	81.9	68.6	38.1	4.5	70.3	1.3	1.3
	WT_3	87,611,003,280	27.9	99.1	16.3	79.2	63.7	30.8	4.2	66.6	1.2	1.1
MG cell line	KNS-42	141,162,591,024	45.0	99.0	27.2	85.1	76.6	53.8	3.8	59.5	1.1	1.0
	G34V-Δ1 (rep1)	75,538,112,152	24.1	99.1	14.8	77.3	57.5	22.0	3.7	56.8	1.2	1.1
	G34V-Δ2 (rep2)	84,711,249,114	27.0	99.0	16.5	79.3	62.2	27.8	3.8	57.5	1.2	1.1
Primary GCTB	GCT02	41,338,157,567	13.2	99.0	7.8	51.9	28.5	7.2	3.9	60.3	1.1	1.0
	GCT06	55,965,792,377	17.8	99.0	10.4	57.7	38.3	14.8	4.1	65.2	1.2	1.1
	GCT09	50,337,616,314	16.0	99.0	9.6	53.7	34.4	12.6	4.2	68.2	1.1	1.0
	GCT11	104,610,140,688	33.3	99.0	18.9	74.9	61.3	36.4	4.0	63.8	1.2	1.2
	GCT23	137,749,991,127	43.9	99.0	24.4	80.2	70.3	48.9	4.2	67.1	1.2	1.1
	GCT30	125,006,758,391	39.8	99.0	23.4	80.7	70.2	47.6	3.8	58.6	1.2	1.1

Supplementary Reference

43. Yokoyama, T., Miura, F., Araki, H., Okamura, K. & Ito, T. Changepoint detection in base-resolution methylome data reveals a robust signature of methylated domain landscape. *BMC Genomics* **16**, 594 (2015)
